# Microfluidic Approaches and Methods Enabling Extracellular Vesicle Isolation for Cancer Diagnostics

**DOI:** 10.3390/mi13010139

**Published:** 2022-01-16

**Authors:** Premanshu Kumar Singh, Aarti Patel, Anastasia Kaffenes, Catherine Hord, Delaney Kesterson, Shaurya Prakash

**Affiliations:** 1 Department of Mechanical and Aerospace Engineering, College of Engineering, The Ohio State University, Columbus, OH 43210, USA; singh.1651@osu.edu; 2 Department of Biomedical Engineering, College of Engineering, The Ohio State University, Columbus, OH 43210, USA; patel.4097@osu.edu; 3 Department of Neuroscience, College of Arts and Sciences and College of Medicine, The Ohio State University, Columbus, OH 43210, USA; kaffenes.1@osu.edu; 4 Center for Life Sciences Education, The Ohio State University, Columbus, OH 43210, USA; hord.63@osu.edu (C.H.); kesterson.18@osu.edu (D.K.); 5 Comprehensive Cancer Center, The Ohio State University, Columbus, OH 43210, USA

**Keywords:** extracellular vesicles, exosome, microfluidics, cancer diagnosis

## Abstract

Advances in cancer research over the past half-century have clearly determined the molecular origins of the disease. Central to the use of molecular signatures for continued progress, including rapid, reliable, and early diagnosis is the use of biomarkers. Specifically, extracellular vesicles as biomarker cargo holders have generated significant interest. However, the isolation, purification, and subsequent analysis of these extracellular vesicles remain a challenge. Technological advances driven by microfluidics-enabled devices have made the challenges for isolation of extracellular vesicles an emerging area of research with significant possibilities for use in clinical settings enabling point-of-care diagnostics for cancer. In this article, we present a tutorial review of the existing microfluidic technologies for cancer diagnostics with a focus on extracellular vesicle isolation methods.

## 1. Introduction

The National Cancer Institute (NCI) defines cancer as a disease in which some of the body’s cells grow uncontrollably and spread to other parts of the body. Solid tumors can spread to other anatomical locations, recur in the same location post-treatment, or invade nearby tissue. It is now generally believed that cancer is caused by changes to genes that control cell function, especially impacting processes on how cells grow and divide. There are more than 100 types of cancer, often named for the organs or tissues where these tumors may form. The treatment of cancer requires reliable diagnostics to identify the type and extent of the disease.

The accepted gold standard for cancer diagnosis is through a tissue biopsy [1]. However, recent reports have noted the multitude of challenges presented by tissue biopsies as these are invasive procedures that may lead to patient discomfort and increase the risk of cancer seeding other locations when carried out on inaccessible tumors [2]. A tissue biopsy provides a snapshot of a tissue sample at a given time with only a small fraction of the suspected tumor extracted for a biopsy [3]. Subsequently, an even smaller fraction of tissue is analyzed, and therefore the tissue biopsy may not accurately portray intratumor spatial heterogeneity [4,5,6].

With continued advances in cancer research [7,8,9], multiregion genetic analysis of consecutive tumors has shown that each tumor presents diverse, spatially distinct mutations with varied phenotypes within the same tumor [6]. Moreover, a single tumor biopsy sample may be inadequate to develop personalized medicine strategies due to the variations in tumor properties [10,11]. Complexity, diversity, and varied physiological locations for solid tumors [12] lead to additional limitations such as not having enough tumor tissue available for biopsy or not being able to monitor intratumor temporal heterogeneity or metastatic sites [5,13].

As a complementary method to tissue biopsies, a liquid biopsy is an investigation and analysis of biofluids to identify biomarkers in a patient fluid sample for cancer diagnosis, prognosis, and monitoring [14,15]. Liquid biopsies rely on the constituent materials in biofluids representing the tumor state. Consequently, the main analytical targets in liquid biopsies include extracellular vesicles (EVs), circulating nucleic acids, and circulating tumor cells (CTCs) [16]. Liquid biopsies may provide additional potential benefits due to relatively low cost [17,18], being minimally invasive [19,20], and providing the opportunity for detailed molecular profiles of tumor-derived materials shed into a variety of biofluids, such as blood [21], urine [22], saliva [23], and cerebrospinal fluid (CSF) [24,25].

Microfluidics is the development and study of devices and systems with operational dimensions in the 1–100 µm range for the manipulation of small (10^−9^–10^−18^ L) quantities of fluids [26]. As technological progress in microfluidics [27] has continued over the past 20 years, the use of analyzing patient biofluids (e.g., blood, urine, or saliva) containing particles sized from 10 nm to 100 µm as a diagnostic tool for cancer has also found major interest [28,29,30]. Microfluidic devices have been extensively used for the isolation, enrichment, and detection of large biomolecules like DNA [31] and proteins [32], as well as extracellular vesicles [33], circulating tumor cells (CTCs) [34], and circulating nucleic acids [35]. The compact nature of microfluidic devices helps conduct multiple unit operations on a single device with integrated functioning [36,37]. These devices are compact and portable and, therefore, suitable for point-of-care (PoC) diagnostics [38,39].

**Figure 1:**
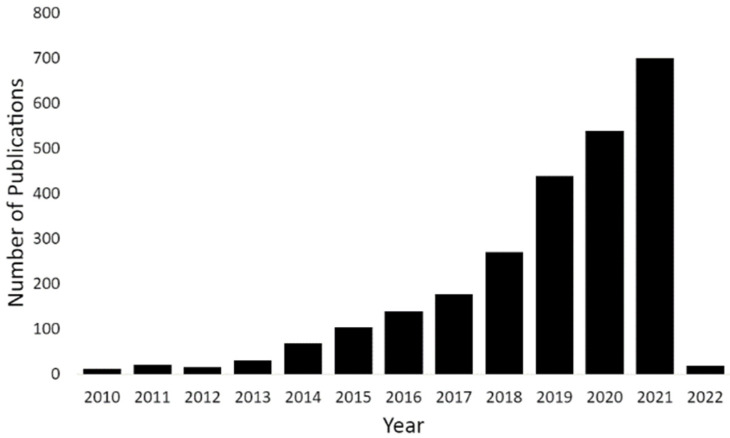
Number of papers published over the last decade with keywords “microfluidics” and “liquid biopsy”. Data obtained from Scopus using a keyword search in December 2021.

The growing interest in developing microfluidics-enabled technologies for exploiting the advantages offered by liquid biopsies is seen in Figure 1, as reflected by the rapid growth of peer-reviewed publications in this area. A search for the keywords “liquid biopsy” in December 2021 in Elsevier’s database Scopus yields 11,918 articles published since 2010. The largest numbers were published most recently in 2020 (2182) and 2021 (2426). Adding the keyword “microfluidics” shows 2520 publications in the last decade, with 2020 showing 537 articles published and the partial year for 2021 already showing 699 articles published, compared to fewer than 20 articles published 10 years ago.

Therefore, the purpose of this article is to reach a broad audience of science and engineering researchers developing new microscale flow-based technologies for cancer diagnostics. This review article focuses on microfluidic and nanofluidic devices and the various technological approaches implemented through these devices for liquid biopsies with an emphasis on methods and approaches used for isolation, detection, and analysis of extracellular vesicles (EVs) from biofluids. The article describes a few conventional isolation techniques to provide a contrast to the emerging microfluidics technologies, with references cited in Section 3 providing the readers with an opportunity to consider conventional EV isolation methods in further detail. Therefore, this article is structured with an overview of the conventional EV isolation techniques followed by a discussion of various microfluidic technologies using phenomena like immunoaffinity, filtration, acoustofluidics, inertial microfluidics, and electrokinetics. It should be noted that there are no standardized definitions to compare the isolation efficiency of a microfluidic device and subsequent sample purity. Each study defines parameters relevant to the study for quantifying the performance of the respective devices; hence, in this review, we have included the definition of parameters as noted by the respective authors. We believe the field as a whole can benefit from a standardized definition and generation of comparative metrics, allowing the comparison of various microfluidic technologies.

## 2. Importance of Extracellular Vesicles

**Figure 2:**
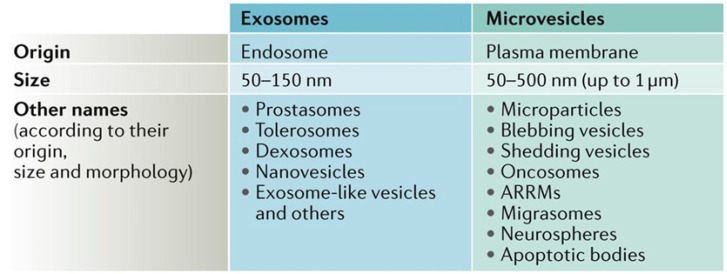
Extracellular vesicles are heterogeneous membrane-bound vesicles that are characterized based on size and origin. Microvesicles are typically larger (100–1000 nm), whereas exosomes are smaller (50–150 nm). There are a variety of terms used to describe extracellular vesicles, with emerging consensus on defining them as small or large EVs. Reprinted by permission from Springer Nature Customer Service Centre GmbH: Springer Nature, Nature Reviews Molecular Cell Biology [46].

Extracellular vesicles (EVs) are a heterogeneous collection of membrane-bound carriers released by nearly all cells [40,41]. EVs carry complex cargoes, including proteins, lipids, DNA, miRNA, and nucleic acids [42,43]. Previously, it was assumed that EVs were a mechanism to discard nonfunctional cellular components [44,45]. Based on size, composition, and origin, EVs can be classified into two main categories: exosomes and microvesicles [3]. Exosomes are small EVs (30–100 nm) and are secreted into the extracellular environment via fusion of multivesicular endosomes with the cell surface, whereas microvesicles are larger EVs (100–1000 nm) that are released by the outward budding and cleavage of the plasma membrane as shown in Figure 2 [46]. Exosomes are of particular importance because these particles have been shown to contain biomarkers, such as nucleic acids and proteins, from their origin cell that have been shown to influence intercellular communication [47]. Further work is needed to evaluate the cargo and roles for microvesicles, especially the large EVs greater than 500 nm in size.

**Figure 3:**
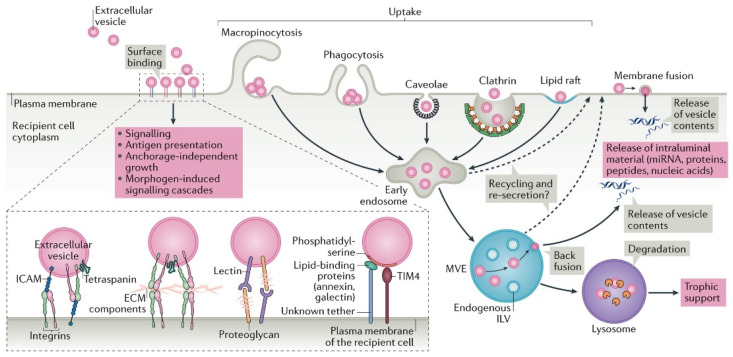
Visual representation of receptor-mediated endocytosis, macropinocytosis, phagocytosis, and membrane fusion of EVs into recipient cells as a means of intercellular communication. Reprinted by permission from Springer Nature Customer Service Centre GmbH: Springer Nature, Nature Reviews Molecular Cell Biology [46].

Through modulation of intercellular communication, EVs also play a role in tumorigenesis [48,49,50]. The tumor microenvironment, which includes blood vessels, fibroblasts, immune cells, and cancer cells, regulates tumor resistance, progression, and metastasis, and all cells within the microenvironment can release EVs [51]. Additionally, researchers have discovered that tumor cells may release more EVs than normal cells [3,52]. Normal or noncancer cells can internalize EVs via receptor-mediated endocytosis, phagocytosis, macropinocytosis, or fusion with the membrane, as shown in Figure 3, resulting in the subsequent changes in the recipient cell [46]. Therefore, tumor-derived exosomes can travel to distal premetastatic target cells and promote metastatic tumor growth by initiating stromal support of tumor angiogenesis, decreasing the antitumor immune response, and enhancing the proliferation of tumor cells [40,53,54,55,56].

For example, in 2018, Guisti et al. demonstrated intercellular EV modulation in vitro by treating normal human dermal fibroblasts (NHDF) with EVs derived from human ovarian cancer cells, SKOV3 (more aggressive) and CABA I (less aggressive) [57]. During treatment, the morphology of fibroblasts transitioned to resemble cancer-associated fibroblasts (CAFs), and this transition was later confirmed by marker analysis found in CAFs, such as α-SMA and FSP-1 [57]. These CAFs were able to influence the proliferation, motility, and invasiveness of surrounding endothelial, tumor, and fibroblast cells, confirming that EVs from ovarian cancer cells cause normal fibroblast cells to behave like CAFs, and these simulated CAFs may alter the behavior of surrounding cells [57]. Similarly, Webber et al. revealed that TGFβ1 expressed on the surface of exosomes derived from prostate cancer cells (PCa) was necessary for the differentiation from normal to tumor-promoting stroma in vivo [58]. In another in vitro experiment, PCa-derived exosomes promoted cell migration, attenuated apoptosis, and escalated cancer cell growth [59].

It is important to note that while the EV cargo often presents a snapshot of the host cell [47,60], the composition of EVs can be different from that of origin cells due to selective cargo sorting [61]. Despite the potential diagnostic and prognostic utility of EVs, the practical relevance of using EVs for routine analysis is limited, as the methodologies required for EV isolation are either time-consuming, provide low yields of EV cargo, or add substantial cost to diagnostic processes [61].

## 3. Conventional Isolation Techniques

Isolation of EVs is briefly described in this section with a particular focus on centrifugation-based methods, ultrafiltration, and polymer-based precipitation methods that have been the cornerstone methodologies for the isolation and detection of EVs.

Table 1 presents a summary of methods for the isolation of cancer-relevant materials using conventional techniques.

### 3.1. Centrifugation-Based Techniques

One of the more common techniques for the isolation of extracellular vesicles (EVs) is ultracentrifugation. In this technique, a sample from cell-cultured media or serum [64] is spun at high speeds, causing a separation of the components to form a pellet containing the majority of the EVs. The literature is inconsistent in defining a precise speed for ultracentrifugation compared to other centrifugation methods [65]. However, generally for ultracentrifugation, spin speeds with accelerations to 2 × 10^5^ *g* have previously been reported [66,67,68,69]. To purify the pelleted samples, it may be necessary to spin the sample multiple times [66]. In standard centrifugation processes, speeds with acceleration of 4 × 10^4^ *g* have been reported [62]. In differential ultracentrifugation, the sample is spun in a sequence of increasing speeds, commonly starting with accelerations of 300–400 *g*, then 2000 *g*, and reaching 10,000 *g* [69].

However, excessive spinning may result in damage to the EVs [66]. On the other hand, inadequate centrifugation may result in a high level of impurities in the EV sample due to co-isolation, which occurs when other components in the liquid samples such as extravesicular protein complexes or aggregates, lipoprotein particles, and other contaminants [70] are incorporated into the isolated EV sample [67]. Impure samples complicate the analysis of EVs as it is difficult to determine whether characteristics are directly related to the EVs or the other co-isolated components [67]. Moreover, past results have also shown that extended centrifugation beyond 70 min can result in higher yields of RNA and protein from the EVs, but duration beyond four hours may result in the presence of excess protein [65]. Consequently, determining the optimal centrifugation conditions for the spin duration is important [65].

Previous reports have evaluated the efficiency of multiple cycles of centrifugation at 4 × 10^4^ *g* versus ultracentrifugation at 11 × 10^4^ *g* in the isolation of EVs, and the results have indicated that five cycles at either acceleration may be required to obtain a suitable sample with the two methods generating similar results [62]. Others have examined the biofluid viscosity dependence on the efficiency of the EV isolation. The results suggest that sample dilution prior to ultracentrifugation may be advantageous [71]. In a comparison of isolation methods, when ultracentrifugation was used in isolation from samples in cell culture media, particle concentrations of 6.20 × 10^8^, 6.33 × 10^7^, and 9.17 × 10^6^ particles/mL were obtained, respectively, over three samples in a concentration gradient [64]. In the same comparison, the use of ultracentrifugation in serum samples produced particle concentrations of 6.35 × 10^9^, 2.22 × 10^9^, and 1.23 × 10^9^ particles/mL over three samples [64]. In a separate study comparing EV isolation methods, the purity obtained through differential ultracentrifugation was found to be 78.2 ± 0.6%, where purity is based on the particle counts before and after treatment of Triton X-100 (used as a non-ionic surfactant for lysing the phospholipid bilayer of EVs) [63]. The drawbacks of using ultracentrifugation were noted throughout this section with the primary ones relating to high cost, low EV yield, and long run times; however, this technique may yield higher protein purity [64].

### 3.2. Size Exclusion

Size exclusion chromatography is another method of EV isolation in which particles are separated based on size. This method can be applied to a wide variety of biofluids, including cell culture media, blood plasma and serum, urine, milk, saliva, nasal lavage, synovial fluid, cerebrospinal fluids, ascites, and tear fluids [72]. A sample is loaded into a column often containing cross-linked but porous agarose beads [72]. Larger molecules pass through the column as they are too large to enter the pores, while smaller components enter the pores and take longer to elute [69]. EVs are larger than smaller molecules [68] and therefore elute faster. One advantage of using size exclusion chromatography is that it has minimal impact on EV properties [72,73], with the sample purity, scalability, and reproducibility offered by this method being considered positives [69]. Work of Tian et al. on size exclusion chromatography performed using qEV columns shows purity of 28.1 ± 0.8% [63] with twenty minutes to process one sample [69,74]. They lysed the phospholipid bilayer of EVs using non-ionic surfactant Triton X-100 and used the particle counts before and after treatment of Triton X-100 as a measure of EV preparations purity [63]. However, this method may not be effective in isolating particles of similar sizes or EVs from lipoproteins [72,75].

### 3.3. Polymer-Based Precipitation Methods

In polymer-based precipitation methods, reagents are added to a conditioned culture medium, causing the lower-solubility components of the sample to precipitate out of the solution [76]. The sample is then spun with accelerations of approximately 1500 *g* [77] to obtain a collection of EVs [69]. This creates a pellet similar to that obtained through the use of ultracentrifugation but avoids the higher centrifugal forces that may damage EVs [78]. These methods have an incubation time from 30 min to 12 h [78]. One disadvantage of this method lies in the fact that co-isolation may occur [78,79]. Additionally, the reagents added are often difficult to remove, and these reagents can interfere with the subsequent analysis of EVs [78,80]. Protein concentration and particle numbers are commonly used for the quantification of exosomes. Generally, exosome purity is defined as the ratio of particle number to protein concentration [81]. Moreover, the purity of these samples may be lower than that obtained by other methods such as ultracentrifugation due to protein contamination [64]. For example, components of plasma such as fibrinogen may interfere with sample purity, but additional steps, including treatment with thrombin and centrifugation, may eliminate this interference [63]. Additionally, serum albumin and apolipoprotein E were found in samples isolated through the use of polymer-based precipitation methods [82]. The purity of two different types of polymer precipitation was assessed, and one produced a purity of 5.3 ± 2.6%, and the other had a purity of 18.5 ± 1.5% [63]. Precipitation methods may be favorable due to the preservation of biological activity found in EVs and the minimal equipment required [76]. However, the commercial kits used in polymer-based precipitation methods use supplies costing 4 USD/mL of the sample [76] and total cost reaching USD 50 per test. To minimize expenses, lower-cost precipitation reagents such as polyethylene glycol may be used [83]. When used alongside ultracentrifugation, these methods may result in higher yields than other conventional isolation techniques [84]. In fact, particle concentrations obtained through polymer-based precipitation methods maybe two to four orders of magnitude larger than those obtained through ultracentrifugation only [63].

## 4. Microfluidic-Based Devices for Extracellular Vesicle Isolation

While much progress has occurred over time in cancer diagnostic methods, the use of EVs remains underutilized, as articulated in the previous sections. The challenges in the isolation and capture of EVs prompted the development of microfluidic systems to separate EVs in a relatively short time (10–200 min) with small sample volumes (100 µL–8 mL) [30]. It is worth noting that cancer diagnostic methods deploying microfluidics and nanofluidics constitute a vast area of research, with circulating cancer cells, cell-free DNA, and other biomarkers also being researched extensively. However, as the focus of this work is on EVs, in the sections to follow we describe only a subset of this broader field, as many other reviews are available for other biomaterials used in cancer diagnostics [5,15,16,85,86,87,88,89].

Table 2 presents a summary of methods for the isolation of cancer-relevant materials using microfluidic technologies.

### 4.1. Isolation Based on Immunoaffinity

In immunoaffinity-based separation, molecules are selectively captured due to specific interactions between antibodies and antigens [104]. The stationary phase often consists of antibodies that have been immobilized and target specific antigens within the sample and isolate portions containing this specific antigen [105]. The implementation of this approach for microfluidic devices usually requires functionalization of the walls of a microfluidics device with immobilized antibodies [104]. Therefore, surface preparation for functionalization [106,107] plays an important role in tethering antibodies [96]. Physicochemical interactions leading to eventual binding or capture of antigens at the immobilized antibody site include hydrogen bonding, coulombic interactions, Van der Waals interactions, and hydrophobic interactions [104].

**Figure 4:**
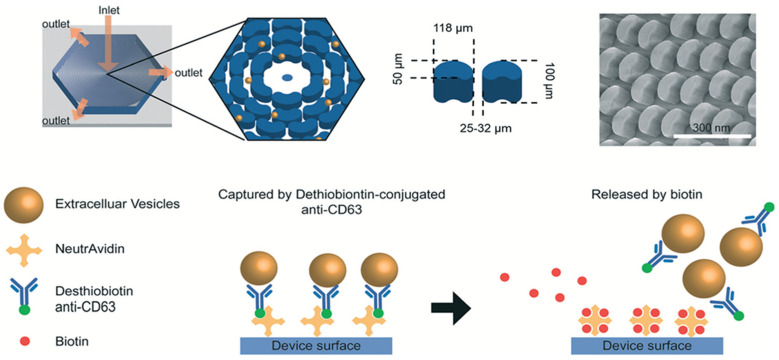
Schematic showing features and functionality of the OncoBean Chip. Width, length, and height of posts were 50, 118, and 100 µm, respectively, with an interpost distance of 25–32 µm. NeutrAvidin is used to coat the surface of the device, which helps in the incorporation of desthiobiotin-conjugated antibodies required for recognition of surface markers of EVs. Biotin is used for the release of the desthiobiotin-antibody-EV complex and effectively allowing for collection of EVs. Reprinted from [108] with permission from the Royal Society of Chemistry.

A high throughput implementation of immunoaffinity separation is the ^HB^EXO-Chip, a device featuring eight channels and a herringbone design that allows for the separation of EVs 30–150 nm in diameter from plasma [90]. The capture efficiency was calculated by allowing 50 million exosomes per milliliter of PBS solution to flow into the device, followed by measuring the concentration of nanoparticles before and after the sample run through the ^HB^EXO-Chip to determine the number of particles captured [90]. The ^HB^EXO-Chip has demonstrated a 75% capture efficiency of tumor-derived exosomes from plasma [90]. Other devices reported for isolating EVs 50–150 nm in diameter have targeted specific cancer biomarkers [91]. For example, a CD-63-1 aptamer was designed for the isolation of EVs 50–150 nm in diameter from tumor samples which are positive for CD-63, considered to be a biomarker in certain types of cancers, including breast cancer [91]. The OncoBean chip (Figure 4) uses biotin-avidin chemistry to facilitate the collection of EVs [108]. Kang et al. reported on the dual-utilization OncoBean (DUO) by targeting separation of EVs using melanoma-specific antibodies melanoma cell adhesion molecule (MCAM) and melanoma-associated chondroitin sulfate proteoglycan (MCSP) [92]. In another implementation of immunoaffinity with microfluidics, devices use magnetic beads coated with antibodies [109]. Additionally, specific biomarkers can also be used to isolate specific EVs with diameters 40–120 nm [110]. For example, Sharma et al. used melanoma-specific biomarkers such as mAb 763.74 (specific for CSPG4 epitope) for the isolation of EVs (30–150 nm) from melanoma cells [93].

### 4.2. Isolation Based on Size

#### 4.2.1. Filtration

Filtration within microfluidic devices is a passive, size-based isolation technique that utilizes a physical barrier to isolate desired EVs. The filters can be microfabricated, or existing filtration media can be integrated within microfluidic devices [111,112]. Filtration systems provide advantages over other microfluidic mechanisms due to their inherent simplicity with minimal requirements to label the desired EV products with fluorescent tags for imaging prior to separation. Some isolation devices utilize common labeling methods like fluorescent tagging to image EVs using fluorescent microscopy, though such techniques can disrupt the entrapment ability by altering the size, shape, and functionality of the molecules to which they are attached. Label-free isolation methods used in filtration devices contribute to higher entrapment efficiency while maintaining the functionality of EVs so that they can be examined after isolation [111]. However, the main challenge for these EV isolation methods is the lack of specificity in isolating particles [96].

**Figure 5:**
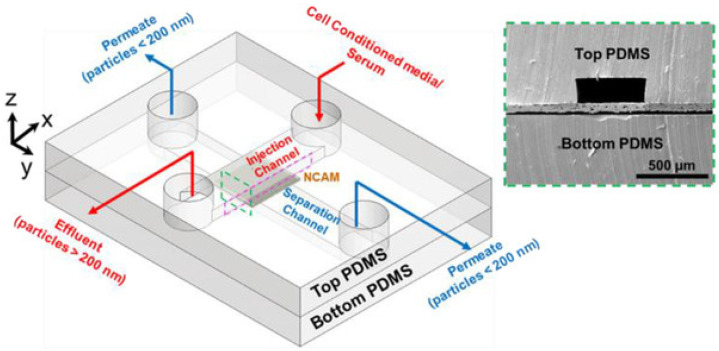
Schematic of Casadei et al. microfluidic filtration device consisting of perpendicular injection and separation channels separated by a nanocapillary array membrane (NCAM). Figure used with permission from [96].

Many implementations of EV isolation using size-based filtration have been reported. For example, Liang et al. fabricated an integrated double-filtration device that isolated EVs from bladder cancer patients [94]. The device consisted of two polycarbonate membranes with 30 and 200 nm pores, which enabled the isolation of EVs within the 30–200 nm size range, and after filtration of the urine samples, the EVs were then detected and quantified using enzyme-linked immunoassay (ELISA) [94]. The isolation efficiency was defined as the ratio of the number of EVs isolated and the number of EVs in the input sample. In comparison against healthy control urine samples, the samples from bladder cancer participants demonstrated a significant increase of EVs present in the urine with 74.2% isolation and entrapment efficiency [94]. To examine its diagnostic effectiveness, the authors determined the device’s sensitivity and specificity, where sensitivity refers to the ELISA chip’s ability to correctly identify cancer-related EVs, and specificity refers to the chip’s ability to accurately distinguish non-cancer-related particles. Overall, the device demonstrated 81.3% sensitivity with a specificity of 90% [94], thus suggesting clinical feasibility for the use of this device for cancer diagnostics. Another device of interest—a ciliated micropillar-based filtration device developed by Wang et al.—successfully isolated exosome-like lipid vesicles from a 30 μL injection sample with high efficiency in 10 min [95]. Smaller, 83 nm lipid vesicles were trapped and recovered within the device with ~60% retention, while larger, 120 nm lipid vesicles observed a 15% retention decrease [95]. Casadei et al. integrated the tasks of size-based separation in a crossflow arrangement with the CD-63 antibody immunoaffinity-based capture of liposarcoma-derived EVs in a single micro-nanofluidic device (Figure 5) and achieved ~76% and ~32% EV recovery for liposarcoma cell-conditioned media and dedifferentiated liposarcoma patient serum, respectively, when compared against ultracentrifugation [96]. They also reported a significant advance over existing state-of-the-art techniques with a five-fold enhancement in the quantity of liposarcoma-relevant EV-DNA obtained in 30 min [96].

#### 4.2.2. Acoustofluidics

Acoustofluidics are microfluidic devices that integrate microfluidics and wave acoustics. These devices use acoustic (or sound) waves for particle patterning, transport, focusing, separation, sorting, and enrichment of particles [113]. These devices use either surface acoustic waves (SAWs) or bulk acoustic waves (BAWs) [114]. Acoustic waves which propagate along the surface of elastic material are called SAWs [115]. In contrast, BAW are standing waves that are generated within the volume of the elastic medium and propagate in the interior of the device [114]. Acoustic radiation and acoustic streaming are the two main forces that govern the separation of particles which depend on the particle size and material properties, including density and compressibility. Acoustic radiation forces are experienced by a particle when it interacts with an acoustic wave and is proportional to the particle volume, whereas acoustic streaming arises in the fluid by the absorption of high acoustic oscillations and induces the size-dependent Stokes drag force on the particles suspended in the fluid [116,117]. Acoustofluidic devices provide various advantages for EV isolation because these devices can be operated label-free with minimal contact and use of reagents [114,118].

Wang et al. developed an acoustofluidic device for the detection of human papillomavirus-associated oropharyngeal cancer (HPV-OPC) using human papilloma viral (HPV) DNA in the whole saliva as a diagnostic means for HPV-POC [97]. Their device consisted of a PDMS microchannel 100 μm in height and 800 μm in width bonded with two pairs of interdigitated transducers (IDTs) generating SAWs at frequencies of 20MHz and 40MHz [97]. The two IDTs were at an angle with respect to the microchannel. The output resulted in isolated EVs in the 30–150 nm diameter range [97]. Their results showed an insignificant effect of variable viscosity (from 1.10 to 2.30 mPa.s) of saliva samples on the number of isolated EVs [97]. In a similar device, Wu et al. exhibited the isolation of exosomes from whole blood [98]. Gu et al. proposed an acoustofluidic centrifuge system capable of nanoparticle transport, concentration, and separation [99]. Their device consists of a circular PDMS containment ring with a pair of tilted IDTs surrounding the circular PDMS [99]. Acoustic radiation force and drag force produced by the SAWs generate a rotational vortex field in the sample droplet, which forced the particles to follow a helical trajectory, resulting in their rapid concentration to the center of the droplet [99].

Ku et al. used acoustic trapping for the enrichment of EVs from urine, cell-cultured conditioned media, and blood from healthy volunteers [100]. Their device output sample carried EVs varying from exosomes to microvesicles in size and included observable levels of intravesicular microRNAs and further confirmed no impact of acoustic waves on the integrity or miRNA content of the trapped vesicles [100]. Lee et al. developed a nanofilter based on acoustofluidics for the separation of extracellular microvesicles and isolated exosomes of diameter less than 200 nm from erythrocyte-derived vesicles from stored blood units and cell-conditioned media with a separation yield of >90% [101].

#### 4.2.3. Inertial Microfluidics

An emerging class of devices examines the size-dependent isolation of neutrally buoyant particles via lateral migration in a non-Newtonian fluid [102,119,120]. Such devices utilize the non-Newtonian viscoelastic properties of blood or saliva to enhance simple isolation capabilities while minimizing pre-isolation modifications [119,121]. Unlike Newtonian fluids, particles in viscoelastic flows are subject to an imbalance of normal stresses that drives their lateral migration, such as the inward driving force of fluid elasticity, the outward force of shear thinning, and particle motion [119,120]. Larger particles demonstrate a tendency to migrate toward the center of the channel at a faster rate, while smaller particles remain along the side walls of the device [102,121]. Such microfluidic devices are advantageous as they do not require externally applied fields (e.g., electric, magnetic, or acoustic), which simplifies device design and fabrication [102].

**Figure 6:**
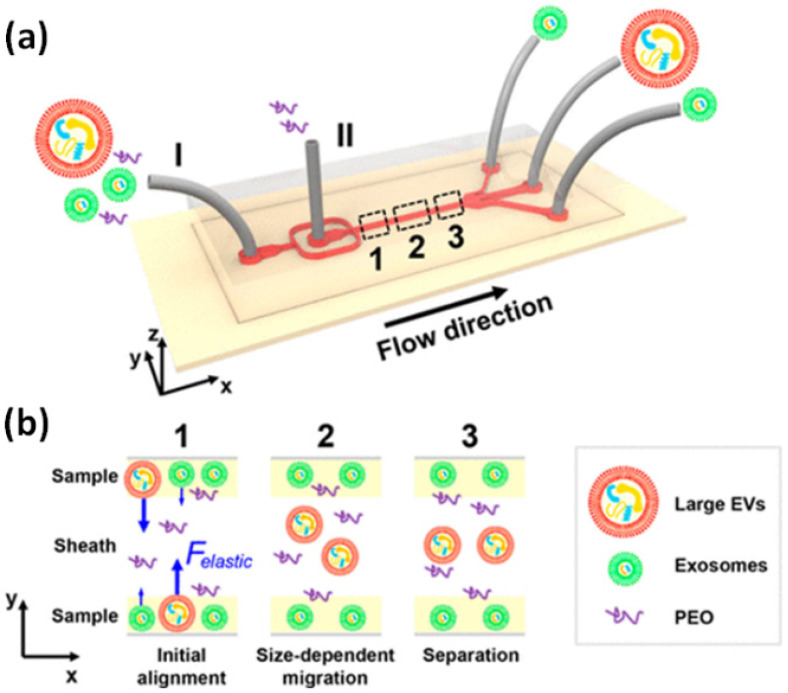
(**a**) Schematic of the microfluidic chip used by Liu et al. [102] for exosome separation from large EVs. The chip consists of two inlets and three outlets where EVs are collected in the center outlet while exosomes exit the two peripheral outlets. (**b**) Illustration of exosome isolation in a viscoelastic medium via elastic lift force (blue arrows) to migrate larger EVs toward the center of the channel while maintaining lateral exosome flow along the side walls of the device. Reprinted with permission from Lui et al. [102]. Copyright 2017 American Chemical Society.

Exploiting fluid inertia and viscoelastic properties has been successful for CTC isolation. However, their use for isolating EVs is limited as with nanoscale particles such as EVs, the inertial lift forces [122] are much smaller, and the standard approaches of these inertial microfluidics may not work. However, recent advances in combined electrokinetic and Poiseuille flow have shown the ability to manipulate dielectric particles in Newtonian flows [123,124], with the application to EV isolation remaining an open question. Though, for non-Newtonian fluids, Liu et al. reported isolating exosomes from other large EVs in a diluted poly-(oxyethylene) (PEO) solution that served to enhance the viscoelasticity of the solution and generate the lift forces responsible for EV isolation from smaller particles (Figure 6) [102]. Nanoparticle tracking analysis (NTA) was used to determine the recovery rate and purity of the separation by measuring the size distributions of the initial and processed samples [102]. The device demonstrated a greater than 90% separation purity with more than 80% recovery [102].

### 4.3. Isolation Based on Electrokinetics

The coupling of an applied electric field to fluid flow gives rise to electrokinetic flows [125]. There are a variety of electrokinetic phenomena [126] that have found use in microfluidics, such as electrophoresis [127], electromigration [128], electroosmosis, dielectrophoresis [129], streaming potential, and sedimentation potential.

Electrokinetic phenomena have also been used for the isolation and detection of EVs [130,131,132,133,134]. Aïzel et al. used a radial geometry in a micro-nanofluidic device for the enrichment of viruses and exosomes [135]. On application of an electric field across 100 nm deep radial channels, they observed concentration and repulsion at the cathodic and anodic part, respectively, achieving an enrichment factor of up to 800 for 50 nm nanoparticles within 1 h [135]. Dey et al. used a symmetric AC electric field [136] in a converging-diverging channel to analyze the trapping of charged microparticles [137]. They studied the trapping mechanisms at low (≤100 Hz) and intermediate (from ~100 Hz to 100 kHz) frequencies and reported a significant dominance of linear electrokinetic phenomena, including electrophoresis and electroosmosis, over the effect of positive electrophoresis in the concentration profile of the analyte which was trapped [137].

A previous report by Kato et al. showed the use of on-chip microcapillary electrophoresis (µCE) and laser dark-field microscopy to demonstrate the correlation between the average ζ potentials of exosomes extracted from six different types of human cell cultures (normal breast epithelial cells (MCF10A), breast cancer cells (MDA-MB-231 [MM231] and MDA-MB-231-luc-D3H2LN cells [MM231LN]), normal prostate epithelial cells (PNT2), and prostate cancer cells (PC-3 and PC-3M-luc-C6 [PC-3ML])) in serum-free media and their cells of origin [138]. They reported a negative shift in the ζ potential distribution of tumor-derived exosomes compared to exosomes derived from nontumor cells [138]. Extending the study for the label-free prescreening of prostate cancer using the same µCE system [138], Akagi et al. analyzed the ζ potential of prostate cancer exosomes and reported a larger ζ potential for cancer-derived exosomes [139]. Akagi et al. also integrated their µCE system [138] with immunoaffinity for the differential protein expression profiling of individual EVs for the detection of overexpression of CD-63 glycoproteins on EVs [103]. They used EVs collected from the culture supernatant of MDA-MB-231 human breast cancer cells and anti-human CD63 antibody and immunoglobulin G (IgG) as EV markers [103]. 

Devices based on microfluidic techniques for EV isolation typically offer faster separation times with smaller sample volumes compared to conventional EV isolation methods. Table 1 and Table 2 give an overview and comparison of the discussed conventional and microfluidic EV isolation methods. As noted throughout the review, each method offers advantages and disadvantages. At present, there are no standard methods to compare microfluidic device performance and conventional methods. Specific definitions arise from individual studies based on samples used, equipment, personnel, cost, and resource availability. There is also a gap in the literature in terms of heterogeneity in EV isolation techniques with no common definitions or protocol for reporting and defining efficiency parameters, including characterization of EV size, composition, and purity across cancer and methodology types.

## 5. Summary and Conclusions

This tutorial review noted the progress in the use of microfluidics-enabled devices for the isolation of extracellular vesicles from a variety of biofluids. The article also summarized comparisons to existing technologies. Clearly, microfluidics can play an important role in developing translational solutions with impact in point-of-care diagnostics for cancer. The survey of publications indicates robust interest in continued device development and technology progress in new and innovative approaches that use a variety of physical phenomena for isolation and analysis of extracellular vesicles.

## Figures and Tables

**Table 1:** Table summarizing conventional isolation techniques for EVs.

Isolation Method	Operating Principle	Advantages	Study	Isolation Efficiency	Throughput
Centrifugation	Spinning results in separation and pellet formation	High purity	Use of multiple centrifugation cycles for exosome enrichment from human serum [62]	-	-
Size exclusion	Particles separated based on size	Minimal impact on size and features	Quality and efficiency assessment of qEV using nano-flow cytometry [63]	67.7 ± 13.1% [63]	-
Polymer-based precipitation	Precipitation of lower solubility components of sample out of solution	Time efficient; requires minimal equipment	Quality and efficiency assessment of ExoQuick isolation kit using nano-flow cytometry [63]	~82% [63]	-

**Table 2:** Table summarizing several microfluidic techniques for isolation of EVs.

Isolation Method	Operating Principle	Advantages	Study	Isolation Efficiency	Throughput
Immunoaffinity	Interactions between antibodies and antigens	High specificity	^HB^EXO-chip for purifying tumor-derived exosomes and establishing miRNA signature in pancreatic cancer with GPC1+exosomes as biomarkers [90]	~75% [90]	-
			Using CD-63-1 aptamer for the isolation of EVs (50–150 nm) from CD-63 positive tumor samples [91]	-	-
			OncoBean (DUO) using melanoma-specific antibodies MCAM and MCSP for exosome isolation [92]	-	-
			Immunoaffinity-based isolation of melanoma cell-derived exosomes from plasma of patients with melanoma with CSPG4-specific mAb 763.74 as biomarker [93]	-	-
Filtration	Difference in particle size population	No need for external actuation; easy to use	Isolation of bladder cancer EVs from urine samples using integrated double-filtration device [94]	74.2% [94]	-
			Isolation of exosome-like lipid vesicles via a ciliated micropillar device [95]	60% (83 nm lipid vesicles), 45% (120 nm lipid vesicles) [95]	3 μL/min [95]
			Isolation and capture of EVs from liposarcoma cell-conditioned media (LCCM) and dedifferentiated liposarcoma patient serum, with MDM2 and CD-63 as biomarkers [96]	76% (LCCM), 36% (dedifferentiated liposarcoma patient serum) [96]	10 μL/min [96]
Acoustofluidics	Acoustic waves	Biocompatibility, versatility, precision, flexibility	Isolation of salivary exosomes from Human papilloma viral (HPV)- associated oropharyngeal cancer patients with HPV DNA as biomarker [97]	-	-
			Isolating exosomes directly from undiluted human blood [98]	82% [98]	4 μL/min [98]
			Nanoparticle enrichment and separation using acoustic centrifugation [99]	-	-
			Acoustic trapping for the enrichment of EVs from cell culture conditioned media, urine, and blood plasma from healthy volunteers [100]	-	10 μL/min [100]
			Separation of exosomes using acoustic nanofilter system [101]	>90% [101]	-
Viscoelastic flow	Imbalance of normal forces in a non-Newtonian medium	Ease of use, no requirements for external actuation, robust performance once operational parameters are optimized	Separation of exosomes from cell culture media and serum of adenocarcinomic human alveolar basal epithelial cells [102]	>80% [102]	200 μL/h [102]
Electrokinetics	Charge of the particle and electrolyte	Strong actuation force due to linear scaling law	On-chip microcapillary electrophoresis for separation of human breast cancer derived exosomes [103]	-	-
